# Supplementary figures and images for: Migraine aura, a predictor of near-death experiences in a crowdsourced study
Source: PeerJ. 2019 Dec 4;7:e8202. doi: 10.7717/peerj.8202 (PMC6898989; doi:10.7717/peerj.8202)

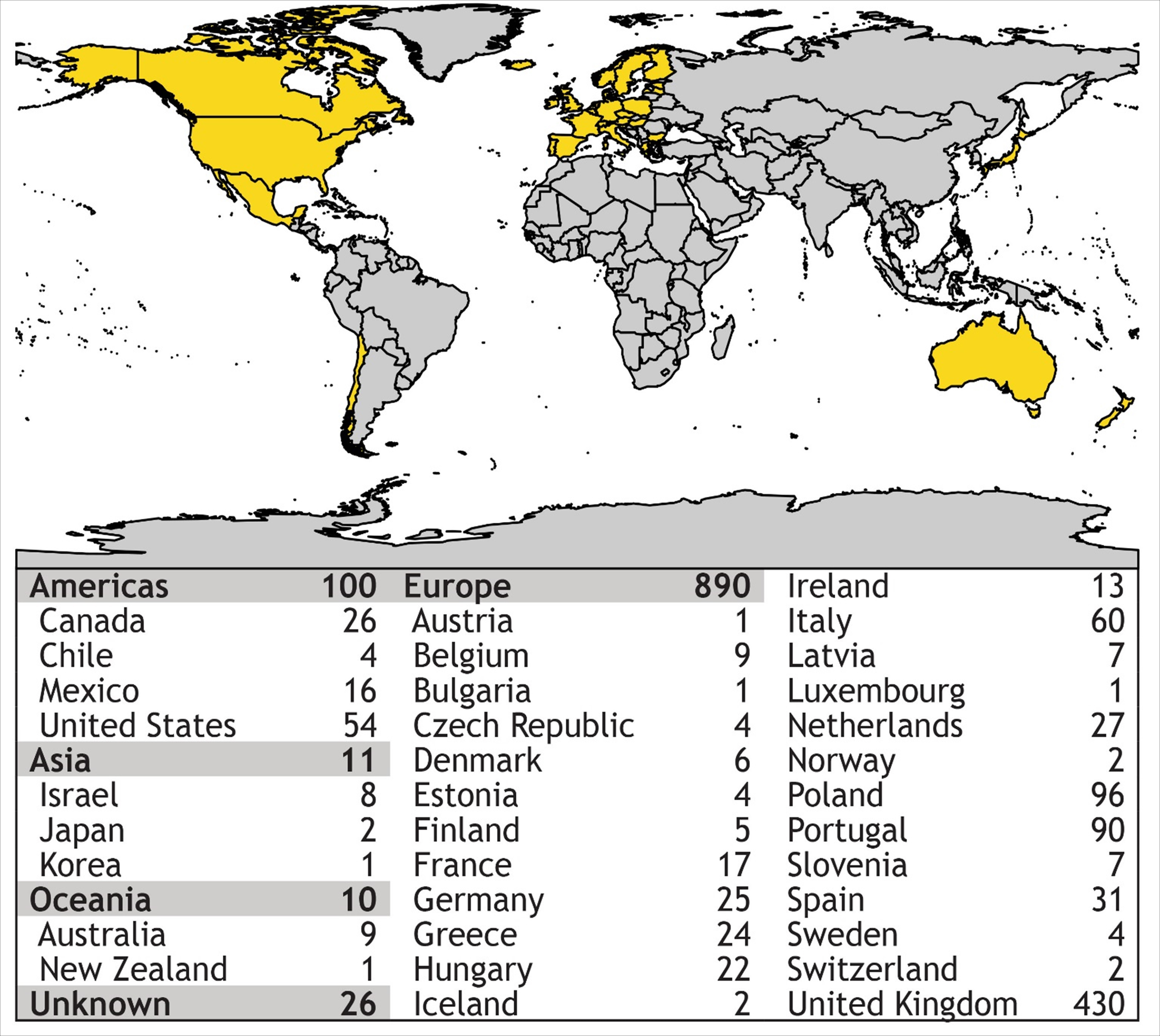

Supplement: Supplemental Information 1 — Using an online crowdsourcing platform, we recruited 1.037 lay people from 35 countries on five continents, the majority from Europe and North America. [file peerj-07-8202-s001.png]
